# Critical events and pivotal factors as tools for analyzing the sustainability of online study programs

**DOI:** 10.1007/s11423-022-10133-9

**Published:** 2022-09-30

**Authors:** Jarkko Suhonen, Erkki Sutinen

**Affiliations:** 1 School of Computing, University of Eastern Finland, Joensuu Campus Yliopistokatu 2, 80100 Joensuu, Finland; 2 Department of Computing, University of Turku, 20014 Turku, Finland

**Keywords:** Sustainability, Online study program, Doctoral studies, Critical factors, Pivotal events

## Abstract

A critical aspect of designing and running online study programs is the identification of factors and elements that could potentially threaten the continuation of studies. In this study, we first identified a set of critical events that occurred in the running of a Finnish online doctoral study program over 16 years. Next, we analyzed the events using a four-pillar sustainability model, which consisted of the economic, social, environmental, and ethical pillars. We detected several contextually relevant and dynamic pivotal factors related to each of the pillars, which had effects on the sustainability of the program at the time of the critical events. The analysis revealed that positive pivotal factors in one sustainability pillar can be used to compensate for negative pivotal factors in the other pillars. Two aspects that were crucial for the sustainability of the online doctoral study program were the resilience and shared commitment of the community involved in its activities, which helped in overcoming any challenges encountered. Based on this study, we recommended that future research should design novel solutions that help online study programs to proactively identify potential critical events and related pivotal factors. Furthermore, studies should find creative approaches for constructively coping with critical events that have been identified.

## Introduction

A critical aspect of ensuring a sustainable design for an online study program is to prepare for potential challenges, problems, and threats to the continuation of studies. In our experience, online learning initiatives start to diminish and eventually fade away when, for example, external funding ends, changes in the leadership of the host institution occur, key people cease to be involved in core activities, or the initiative fails to renew or no longer fits the strategy of the host institution. Research has identified that quality assurance lists, critical success factors, risk analysis tools, and good practices support the sustainable design of online study programs (King & Boyatt 2014; McPherson & Nunes, 2006; Sridharan et al., 2010). However, according to our experience, most existing models do not cope well with unexpected problems and dynamic challenges caused by contextual factors, or even global crises such as the COVID-19 pandemic. Furthermore, existing models rarely consider ethical aspects to be a crucial component of online study programs’ sustainable design. In a contemporary digitalized society, unexpected or unforeseen incidents, changes in circumstances, and the complexity of reality can damage potentially fragile components of online study programs. Thus, a need exists to establish solutions that help program designers to prepare for surprises and unanticipated events. This study contributes to the knowledge base for the sustainable design of online study programs, as we retrospectively analyzed and critically reflected on our experiences of designing, managing, and running an online doctoral study program in educational technology in Finland (Willis, 2009). We anticipate that our work, based on 16 years of working with online doctoral studies, will assist designers, coordinators, and educators in coping with critical events (CEs) and pivotal factors related to the design and running of online study programs.

In this study, we first identified a set of CEs of a Finnish online doctoral study program. Second, we analyzed the identified CEs with a four-pillar sustainability model, which consisted of the economic, social, environmental, and ethical pillars (Suhonen & Sutinen, 2014). Finally, we identified various pivotal factors connected to those CEs. The positive pivotal factors supported the sustainability of the program, whereas the negative pivotal factors posed threats to its sustainability. We also demonstrated that positive pivotal factors in one sustainability pillar can be used to overcome negative pivotal factors in the other pillars. For example, contributions from committed key academics (a positive pivotal factor in the social pillar) can be used to compensate for problems caused by scarce financial resources (a negative pivotal factor in the economic pillar).

The remainder of this paper is organized as follows. In the second section, we discuss what sustainability means in the context of online learning. We also define two main constructs related to our work, namely CEs and pivotal factors. In the third section, we introduce the four-pillar sustainability model, which we used to analyze the CEs and pivotal factors. In the fourth section, we introduce and provide key statistics regarding the Finnish online doctoral study program. In the fifth section, we present a sustainability analysis on the doctoral study program with the four-pillar sustainability model, in which we identified and analyzed a set of CEs and pivotal factors connected to the events. Finally, in the sixth section, we reflect on the results of the analysis, provide design guidelines for practitioners, and discuss future research opportunities.

## Background

### Definition of an online study program

We defined an online study program as a formal education program in which students can complete a full degree or part of it, or receive a formal certificate, by studying mainly online. Online study programs often follow a mixed-model learning and teaching approach, in which face-to-face and online learning solutions, different technologies, and various instructional approaches are mixed and combined to implement pedagogically meaningful and practically relevant solutions to support learners in their studies (Köse, 2010; Sharma, 2010). Online study programs aim to offer flexible ways to study a degree or acquire a certificate since the studies are not tied to a specific physical location (King & Boyatt, 2014). According to Kozar et al., (2015), online study programs offer students opportunities to be self-guided and proceed with their studies without being sidetracked by others. In addition, the use of mixed-mode learning and teaching approaches has the potential to minimize some of the challenges related to “pure” online learning, such as communication problems, feelings of isolation, unhappiness toward independent studying, and separation from the community of practice (Diep et al., 2017; Kozar et al., 2015).

### Sustainability in online study programs

According to Hopwood et al., (2005), the concept of sustainable development combines awareness of the global links between mounting environmental issues, socioeconomic problems related to poverty and inequality, and concerns regarding the future of humanity. Brundtland’s report titled “Our Common Future” provided the following definition of sustainable development: “*Sustainable development is a development that meets the needs of the present without compromising the ability of the future generations to meet their needs”* (Brundtland, 1987). The original sustainability dimensions proposed in the Brundtland report are economic growth, environmental protection, and social equality. However, various definitions and meanings of sustainability exist (Hopwood et al., 2005; Stepanyan et al., 2013). For instance, the United Nations (UN) Global Compact Cities Program released a sustainability model called Circles of Sustainability, which is used to assess and manage projects that aim to attain sustainable outcomes. The Circle of Sustainability model consists of four circles: economics, ecology, politics, and culture (McCarthy et al., 2010). The UN has also defined a set of Sustainable Development Goals (SDGs), which are meant to be blueprints for achieving a sustainable future for all (UN SDGs, n.d.). The SDGs address a set of global challenges related to poverty, education, inequality, climate change, environmental degradation, peace, and justice. One of the SDGs is to achieve universal access to quality higher education, which can provide an ethical motivation for starting to design and implement online study programs.

Our interpretation of sustainability in the context of online study programs was based on the following challenge: How can an online study program survive the opportunities, challenges, and changes that occur? In other words, sustainability can be considered to consist of procedures, practices, and aspects that ensure the continued viability of the online study program (Nicholas, 2008; Sridharan et al., 2010).

Relevant research has identified various quality assurance lists, critical factors, recommendations, and good practices for online study programs, which can be used as design principles for implementing successful and sustainable online study programs (King & Boyatt, 2014; McGill et al., 2014; Nichols, 2008; Salim, 2007; Sridharan et al., 2010; Sun et al., 2008). For example, according to McPherson and Nunes (2006), leadership, structural and cultural issues, technological perspectives, and delivery solutions are crucial to consider when designing online learning programs. Furthermore, Porter & Graham (2016) demonstrated that appropriate infrastructure, technological and pedagogical support, evaluation data, and the institution’s purpose for adopting online learning significantly influence its adoption by faculty. In Table 1, we have synthesized a set of design aspects related to the sustainability of online study programs from the following perspectives: (1) basic requirements, (2) indication of threats and barriers, and (3) strategies for mitigating sustainability risks.

**Table 1:** Sustainability of online study programs

Design aspects	Basic requirements	Indication of threats and barriers	Strategies for mitigating risks	References
Institutional culture	Readiness and openness for online learning	Unrealistic time and work effort requirements for staff and learners Institution is not ready for online learning	Constant development of the institutional culture to support the adoption of online learning	Nichols (2008); McPherson and Nunes (2006); Bhuasiri et al. (2012)
Professional development	Training available for staff on online learning and the use of contemporary learning technologies	Staff training is missing or poorly implemented	Investments in staff training and up-to-date training content	McGill et al. (2014); Sridharan et al. (2010); McPherson and Nunes (2006); Porter and Graham (2016)
Financial and other resources	Sufficient resources and support available from the host university	Lack of support from leadership and management	Strategic ownership and support from management Other people within the organization join the implementation Identification of the true costs of online learning	Sridharan et al. (2010); Nichols (2008); Neely and Tucker (2010)
Technology	Both basic and specific solutions available to support the design and implementation of online studies IT support swiftly available	Insufficient infrastructure and technological support and ineffective use of learning technologies Instructors’ negative attitudes toward online learning and learning technologies	Design and promotion of user-friendly and easy-to-use learning technology solutions Meaningful use of technologies to support the chosen pedagogical strategies Combined top-down and bottom-up approaches for integration with existing systems and practices Use of technologies to meet communication and interaction requirements	McPherson and Nunes (2006); Porter and Graham (2016); Barber et al. (2016); Selim (2007); Bower et al. (2015); Jaggars and Xu (2016); Sun et al. (2008)
Instructional design and pedagogy	Teachers can use various teaching approaches and pedagogical solutions	Standardized teaching approaches Institutional regulations or country’s educational system constrain the use of teaching approaches and pedagogical solutions Lack of pedagogical support	Adoption of flexible pedagogical solutions suitable for a given course/unit Proactive involvement of course instructors: personal touch with learners, prompt feedback, and social presence Designing for active learning Frequent and effective student–instructor interaction	Johnson et al. (2008); King and Boyatt (2014); McGill et al. (2014); Rovai and Downey (2009); Bower et al. (2015); Jaggars and Xu (2016); Sun et al. (2008)

When analyzing the design principles of online study programs from the perspective of sustainability, some common characteristics arise. Specifically, these programs often require the commitment of leadership, organized technological and pedagogical support, continuous evaluation and assessment, and strategic alignment of the program with the institutions’ mission and goals. This means that online study programs are usually highly organized and carefully planned, and they have an institutional, top-down orientation. Observations of the shortcomings of massive open online courses (MOOCs), such as high dropout rates (Chen & Zhang, 2017), reveal a similar phenomenon: teachers are strongly encouraged to use MOOCs in their work, but their hesitation is observed in outcomes that seriously fail to meet the expectations set by the university leadership.

Notably, the problems faced with any top-down designed program is confidence of the institutions in the eternal continuation of the status quo and the ability to plan for predictable conditions. Therefore, the perspective of sustainability, with its origins in the creeping catastrophes that were feared to escalate following the long-term abandonment of global problems, such as poverty and environmental indifference, is relatively unknown to the foundation of these programs. The recent COVID-19 pandemic has confirmed the world’s unpredictability as well as the vulnerability of top-down strategies. Sustainable programs require the commitment, inspiration, and continuous contribution of faculty members which are more critical than those of their leaders. Thus, this much-needed resilience, which presupposes a bottom-up orientation, has been largely missing from the design architectures of online programs. Perhaps, more profoundly, the omission of an ethical perspective from the original set of sustainability dimensions (i.e., economic growth, environmental protection, and social equality) might reduce or limit sustainability to rigid design rules, strict regulations, or quality handbooks.

## Analyzing the sustainability of online study programs—the four-pillar model

In our previous study, we introduced a four-pillar model for analyzing the sustainability of online study programs. The model consists of four sustainability pillars: economic, social, environmental, and ethical (Suhonen & Sutinen, 2014). The first three pillars are our interpretations of the Brundtland Commission’s work on sustainable development (Brundtland, 1987), while our introduction of the ethical pillar to the sustainability model was inspired by the work of Heinonen (2012). The four pillars are described in detail in the following subsections.

### Economic pillar

The economic pillar refers to an online study program provider’s capability to sustain its resources to support the implementation of the program. The typical aspects related to the economic pillar are funding and other available financial resources (Nichols, 2008). According to Stepanyan et al. (2013), crucial aspects of economical sustainability include finding a proper input–output balance, having a profitable business model, and efficiently using available resources. A range of methods have been introduced to support economic sustainability in online study programs, including reducing completion times, identifying hidden costs, and increasing tuition fees (Neely & Tucker, 2010; Rovai & Downey, 2009; Stepanyan et al., 2013) claimed that economic factors are considered most when sustainability in online learning is discussed, such as how online study programs are economically viable or even how they generate profit for the host institution, regardless of other perspectives.

### Social pillar

The social pillar covers the internal dynamics of the individuals involved in the design and implementation of an online study program. It also includes community aspects, which refer to the connections and interactions between the individuals (Stepanyan et al., 2013). According to Russel (2009), interpersonal chemistry and the community’s ability and capability to cope with challenging situations are relevant for social coherence. Furthermore, the promotion and encouragement of diversity, transparent decision-making structures, and a positive attitude toward personal development are relevant to the social pillar. Gunn (2010) claimed that a common vision shared by the community is a positive aspect, whereas conflicting interests and perspectives within the community pose a threat to sustainability.

### Environmental pillar

The environmental pillar deals with how the online study program’s environment—mainly its physical, technical, cultural, and psychological contexts—supports and accommodates studies. The physical-technical context can be called a learning place, while the cultural-psychological context can be called a learning space (Spector, 2015). The environmental pillar also includes learning technologies and other digital technology solutions available for the implementation of online studies. Gunn (2010) and Rovai and Downey (2009) have argued that supportive internal structures, including faculty development, are critical when implementing an online study program. Spector (2015) identified organization atmosphere, technology life-cycles, and support personnel as important environmental aspects. Finally, the environmental pillar could also include outside factors, such as institutional strategies and national governmental agendas. Thus, a sustainable online study program should be tightly integrated both internally into the local environment and externally into the surrounding society and global environment.

### Ethical pillar

The ethical pillar reflects the values behind the goals and the working principles of an online study program. As an example, we present two different ethical perspectives: teleological and deontological ethics. Teleological ethics guide thinking and decision making through the expected value of the actions, as in the principle that the outcome justifies the means (Frankena, 1973; Macdonald & Beck-Dudley, 1994). In the education field, this could mean that expected quantifiable results, such as degrees, guide the activities. Deontological ethics refer to an understanding of the ethical value being based on certain features of the activity itself, rather than on the value it brings into existence (Frankenna, 1973; Macdonald & Beck-Dudley, 1994). An example of deontological ethics is the Golden rule: do to others that which you want them to do you. Furthermore, the principles of beneficence are relevant to ethical decision making: (1) one ought not to inflict evil or harm; (2) one ought to prevent evil or harm; (3) one ought to remove evil or harm; and (4) one ought to do or promote good (Frankenna, 1973; Macdonald & Beck-Dudley, 1994). In the education field, this could mean that personal growth, respect of diversity, or equal access to learning are the driving forces behind the activities.

### Connections between the four sustainability pillars

**Fig. 1:**
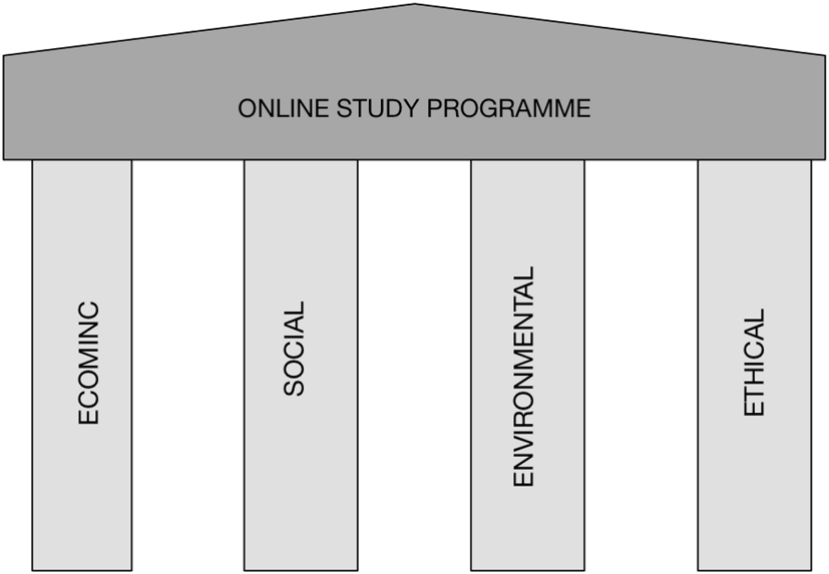
Sustainability pillars

In the four-pillar sustainability model, the pillars are mutually interrelated and even traded off. For example, a trustful work community can function sustainably (even with limited or decreasing financial means), whereas a highly demanding or even threatening work community can endanger sustainability (despite ample financial resources). A constructive tension between the pillars supports sustainability, whereas conflicts between them could scar and damage even a successful online learning initiative. Thus, it is crucial to find a mutually constructive interplay between the four pillars, since relying too heavily on a single pillar or even two could be potentially problematic for the sustainability of the program, as illustrated in Fig. 1.

However, two aspects affect the importance and relevance of the pillars. The first is the scale of a study program, which can vary from a set of individual courses to certificate programs and full degree programs. Completely online universities already exist, as do consortia of online universities. The scale of the online program is visualized by the size of the roof in Fig. 1. Individual pillars can hold a small roof, but the importance of well-balanced support from all pillars grows as the size of the roof increases. The second aspect is the complexity of the program, which exerts stress on the pillars. For example, a university consortium is a highly complex entity, imposing significant demands on all pillars. However, a small-scale online study program can also be complex depending on the contextual factors, such as the study content, diversity of students, teacher qualifications, and learning goals.

### Critical events and pivotal factors: tools for sustainability analysis

We proposed two constructs for analyzing the sustainability of online study programs using the four-pillar model: CEs and pivotal factors. Mertova & Webster (2009) defined a CE as an unplanned and unstructured event that significantly affects the practice of an academic community, which in our case was an online study program. For example, a key staff member leaving the study program could cause serious problems for its sustainability. CEs are often but not always context-dependent, and they can occur unexpectedly, causing serious or even uncontrollable problems for the program’s sustainability. Thus, a CE can be foreseen but not controlled. Furthermore, a CE will typically involve several pivotal factors that illustrate its impact on the sustainability of the online study program. Positive pivotal factors have a constructive effect on sustainability, which increases the possibility of a successful program implementation (Kira & Saade, 2006; Salim, 2007). By contrast, negative pivotal factors can threaten the sustainability of the program, leading to an unsuccessful implementation and ultimately program failure (Kira & Saade, 2006; McGill et al., 2014).

## IMPDET online doctoral study program

### Background

IMPDET (International Multidisciplinary PhD Studies in Educational Technology) is an international online doctoral study program designed and hosted in Finland. The program has been running since 2004, and it was originally designed to provide doctoral studies to applicants from emerging and transitional countries. In the IMPDET studies, doctoral degrees can be completed either in computer science or education. The decisions regarding the discipline and eventually the faculty where the doctoral studies are pursued are primarily made based on the applicant’s previous academic degree. The application procedures, study requirements, research cultures, and supervision arrangements vary between the two faculties, which means that the study experiences of the doctoral students also differ depending on the faculty in which the studies are pursued. However, some students have supervisors from both disciplines, which supports the multidisciplinary nature of their research work.

All doctoral degrees in Finland are research-based, which differs for instance from the USA, where PhD and Ed.D degrees clearly serve different purposes (Dawson et al., 2011; Shulman, 2005) . Moreover, doctoral degrees in Finland are not professionally oriented, as they might be in, for example, the UK. In Finland, to receive a doctoral degree, a doctoral student must (1) complete their postgraduate studies according to the requirements of the university where the degree is completed; (2) demonstrate independent and critical thinking; and (3) write a doctoral dissertation and defend it in public.

An eligible doctoral dissertation in the IMPDET studies can be a monograph or an article-based dissertation, but the preference is for students to write an article-based dissertation. An article-based dissertation consists of two components. First, it includes a collection of scientific publications or manuscripts, which are usually co-authored in collaboration with supervisors, peers, master’s students, or other research collaborators. The article-based dissertations in this program typically include five to seven publications, one of which is an unpublished manuscript. The doctoral student must be the first author in most of the dissertation publications. Second, an article-based dissertation includes a summary section that tells a coherent story of the dissertation study, usually by introducing and motivating the research topic, discussing relevant literature, providing information about the research methods used in the dissertation study, describing and interpreting the main results and outcomes, and critically discussing the scientific contributions and relevance of the dissertation. At the end of their studies, the student selects together with the supervisors the publications that will be included in the dissertation and writes the summary.

Most monograph dissertations in the study program have been hybrids between a pure monograph (unpublished research) and an article-based dissertation. At the end of their studies, the student writes a monograph but refers to the scientific publications in the dissertation’s main body of text instead of including the publications as part of the dissertation. In the program, the main reasons for choosing a monograph dissertation are that the scientific publications related to the dissertation do not form a single coherent research study, there is no clear connection between the individual publications, or the dissertation itself includes an independent scientific contribution supported by the publications.

### Pedagogical components

The pedagogical components of the program are designed to mentor the students through the process of becoming a doctoral-level scholar (Kumar & Coe, 2017) as well as to integrate students into the staff, study program, and host university (Rockinson-Szapkiw et al., 2016). Furthermore, the pedagogical components aim to provide structured support as well as emphasize interaction and communication with supervisors to prevent dropouts (Hart, 2012; Lee & Choi, 2011).

The first and most critical pedagogical component in the program is to provide the doctoral students with an independent research experience. Each student is required to pursue an original and independent study mentored usually by two or three supervisors, but in some cases even team of four supervisors. The students are encouraged to find a research topic connected to a real-life situation, challenge, or problem that emerges from their own cultural or social context. The local relevance and practical aspect of research work was one of the key motivations for launching this program. Furthermore, another study demonstrated that doctoral programs embedded in practice can positively affect doctoral students’ professional growth (Kumar & Dawson, 2014).

The doctoral students in the program can freely use any suitable research approaches and data collection methods for their dissertation study. However, in recent years, action research (Avison et al., 1999; Baskerville, 1999) and design science research (Hevner et al., 2004; Johannesson & Perjons, 2014) supported with mixed-methods data collection have been the most commonly used research approaches. Following the principles of design thinking, the students often design, implement, and evaluate a concrete solution to solve an educational or social problem (Bjögvinsson et al., 2012). For example, a recent dissertation topic concerned developing a mobile learning system to support the teaching of university-level mass courses in the Nigerian higher education context. As part of their research work, the doctoral students are also expected to co-author scientific publications to be published in conference proceedings, scientific journals, or book chapters following a double-blind review process. They are instructed to publish in publication channels that have been classified in the Finnish Publication Forum rating and classification database (https://www.julkaisufoorumi.fi/?lang=en). The publishing requirement is aimed at ensuring the quality of the doctoral students’ research work, since they can ultimately use the publications as part of their dissertation.

The second pedagogical component of the program is the mixed-model teaching and learning approach, which grants students flexibility in pursuing their studies depending on their life situation, the availability of funding, and other personal factors (Bower et al., 2015; Diep et al., 2017; King & Boyatt, 2014). Studies can be conducted (1) almost completely online, (2) completely on campus, (3) or a mixture of campus and online study. While studies can be pursued online, the doctoral students are encouraged to visit the campus to receive intensive supervision, complete course work, and establish contacts with the local research community at the host university. At the end of the studies, the doctoral students are required to defend their doctoral dissertation publicly at the campus of the host university. However, during the COVID-19 pandemic, students have also defended their dissertations online using the Zoom and Lifesize video conferencing systems. Basic tools for online learning have been used in the program to support the mixed-model teaching and learning approach; for example, Wikis and various other online platforms at the host university have been used to share instructions, provide access to research resources, and enable collaboration. In addition, a Yammer group site and a Facebook page have been used to distribute news and information relevant to the studies. Furthermore, Moodle LMS has been the main platform used for course work, while Zoom and various other video conferencing tools have been used for supervision, communication, and online participation in activities.

The program’s third pedagogical component is course work, which supports students’ doctoral studies and research work. The students complete course work worth 30 or 50 ECTS (European Credit Transfer and Accumulation System) credits during their studies. At the host university, 1 ECTS credit equals approximately 26 h of study or required work time. At the beginning of the students’ studies, they together with their main supervisor create an individual postgraduate study plan based on the requirements of the faculty in which they have enrolled.

Three different course work components can be included in the postgraduate study plan. First, the students can study online, mixed-method, and campus-based courses offered by the host university or organized by another university. The topics covered by the courses offered specifically to the students on the study program have included the following: (1) contemporary technologies in education, (2) research methods in computing, (3) the design of smart learning environments, (4) learning analytics, and (5) ICT for development. Furthermore, the graduate school of the host university offers online, mixed-model, and campus-based courses on transferable skill studies, such as scientific writing, scientific presentation, research project management, research ethics, and English for doctoral studies. The courses are available to all doctoral students at the host university, so they are not specifically targeted at IMPDET students. The only obligatory course for all doctoral students in the program is the research ethics course. The students can also complete relevant courses (e.g., MOOCs) outside of the host university, which provides additional flexibility for the studies. The students’ main supervisor or the coordinator of the study program accepts the inclusion of the courses outside of the host university.

The second course work component is individual learning tasks, which provide flexibility and additional opportunities to complete the credit points beside the formal courses. The individual learning tasks are agreed with the main supervisor or the program coordinator. The tasks typically include the following types of activities: (1) orientation at the beginning of the studies; (2) different types of essays or other written assignments; (3) a literature review related to the students’ research topic; (4) project work activities, such as the implementation of a prototype or design tasks; and (5) presentations in scientific conferences. Furthermore, the main supervisor or the coordinator of the program can accept basically any activity or task if it is relevant for the students’ research work or supports their doctoral studies in a meaningful way.

**Fig. 2:**
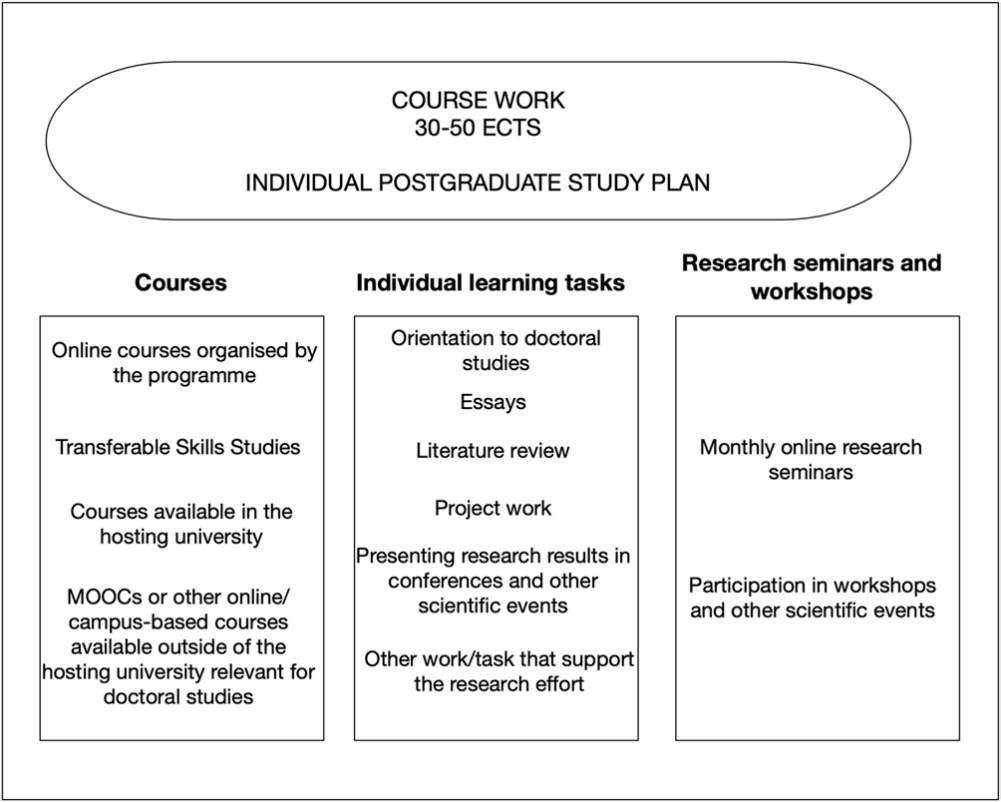
Overview of the course work components

The third course work component is research seminars and workshops. The study program runs monthly online research seminars and students can gain credit points by participating in them and giving presentations. In addition, the program offers irregular intensive scientific workshops, such as those related to Academy of Finland- or EU-funded projects of the research groups involved in the program activities. Figure 2 summarizes the course work components of the program.

The completion time for the postgraduate course work and even the course plan varies between the students; therefore, there are no exact instructions for the students regarding when to complete their course work. The only binding requirement is that at the end of the studies the completed course work should fulfill the minimum requirements set by the students’ faculty. It is also highly common for the original individual study plan accepted at the beginning of the studies to be updated at the end of the studies, since some originally planned courses might no longer be available or new opportunities to complete the course work may arise.

### Key statistics of the study program

At the end of 2021, a total of 44 doctoral students representing 20 different nationalities were enrolled in the program. The first students were admitted in 2004, and most of the doctoral students (over 94% of those admitted) have studied computer science, which is because the program has mainly been run by an educational technology research group of the computing department at the host university. While the program was originally designed and implemented in close collaboration with educational scientists at the host university, this collaboration has diminished over the years.

The first doctoral degree of the program was awarded in 2007, and the first doctoral student to have conducted most of his studies through distance learning graduated in 2008. A total of 32 doctoral students graduated from the program between 2004 and 2021. The average completion time for a doctoral degree is 5 years and 6 months, and the median completion time is 4 years and 11 months. The standard deviation is 2 years and 4 months, which indicates high variation among completion times. There are students who have completed their studies within 4 years (the estimated time for completing doctoral studies in Finland when studying full-time), but several other students have required 9–10 years to complete their studies.

**Fig. 3:**
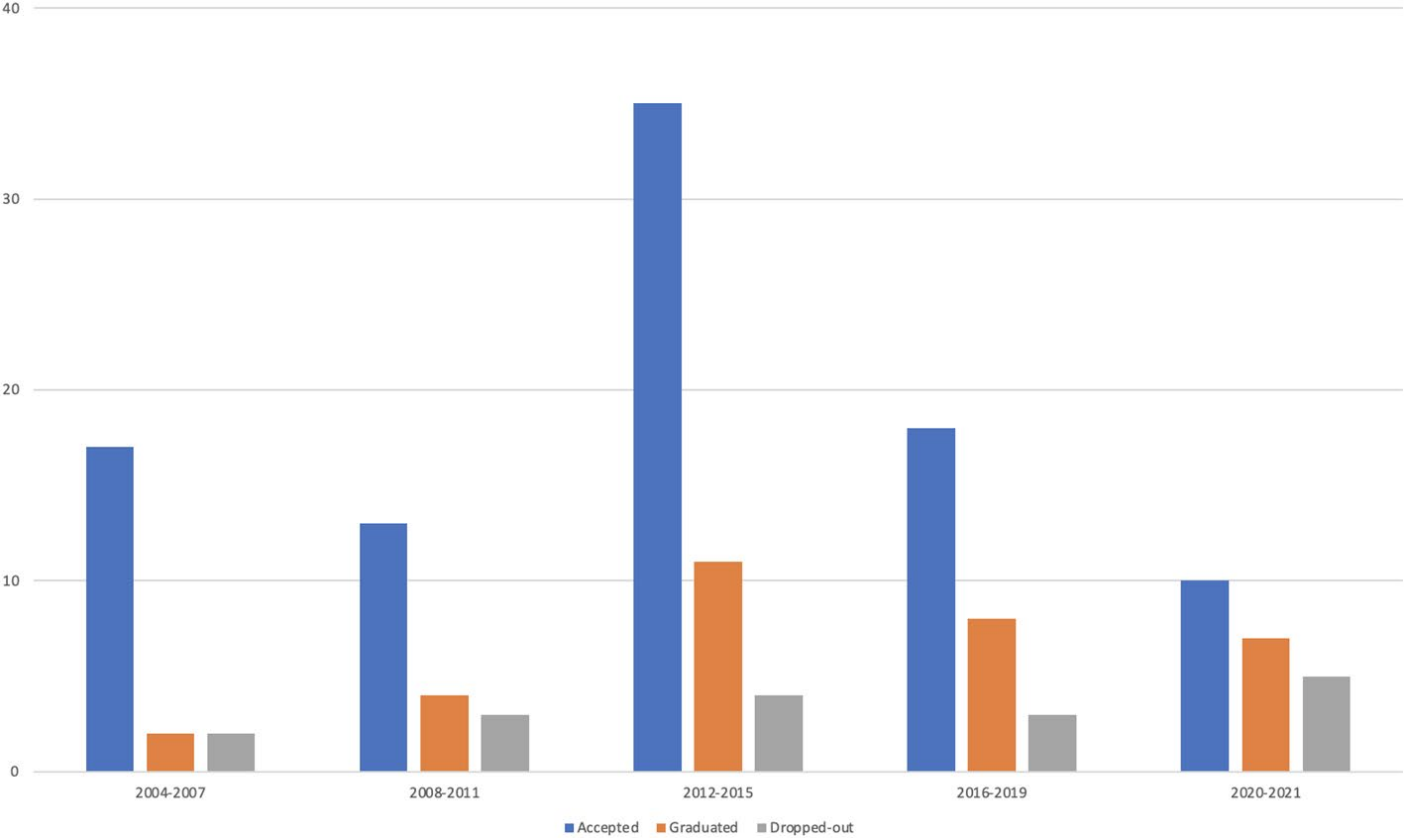
Key student number indicators

Figure 3 presents the key student number indicators of the study program divided into five periods: 2004–2007, 2008–2011, 2012–2015, 2016–2019 and 2020–2021. The figure indicates that during 2012–2015, the number of admitted students was considerably higher compared with other periods. Figure 3 also indicates that the graduation numbers have continuously increased. A total of 93 students have been admitted to the program by end of 2021, 32 (34%) of whom graduated and 17 (18%) officially quit their studies between 2004 and 2021.

The persistence rate of the program has been relatively high compared with, for example, the high attrition rate in a related program in Sweden (Frischer & Larsson, 2000). There are three context-dependent reasons for this phenomenon. First, doctoral students in Finland do not need to pay study fees. Second, there is no upper time limit for the duration of their studies. The flexibility of doctoral studies in Finnish higher education institutions enables doctoral students to study part-time and proceed at their own pace. The main reason doctoral students drop out of the program is moving to another university. Another common reason is if students have not been able to independently pursue doctoral studies through distance learning.

**Fig. 4:**
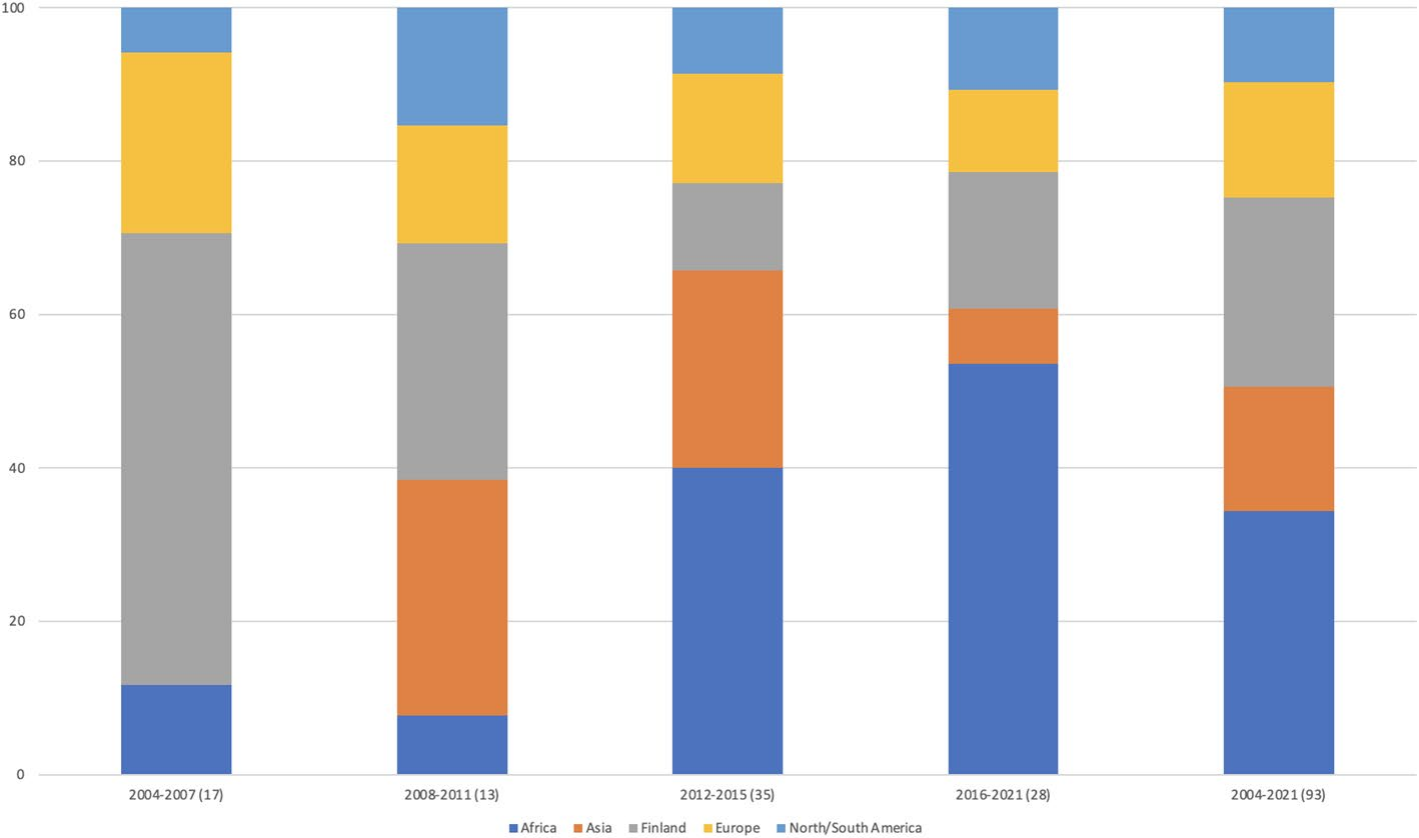
Origin of the admitted doctoral students

Figure 4 presents the origin of the doctoral students admitted to the program. The figure first displays the origin distribution divided into the same four time periods as in Fig. 3, whereas the last bar displays the overall distribution from 2004 to 2021. As seen in the figure, initially most students were from Finland, but gradually more students have joined from outside of Finland, especially Africa. During 2012–2021, the majority of admitted students have been from Africa.

## Sustainability analysis of the IMPDET online doctoral study program

### Identifying the critical events

We began our study by identifying and describing a set of CEs of the program during 2004–2021. As stated above, a CE refers to any circumstance that allows for or threatens the continuation of an online study program. In the next step, we narratively analyzed the identified events using the four-pillar sustainability model. A narrative inquiry is based on human stories of experiences, and the method has been used in a variety of disciplines, including education and computing (Mertova & Webster, 2009). Narrative inquiry can be used to investigate how humans experience the world holistically. Our analysis also included aspects of autoethnography since we based it on personal reflections on our own experience (Ellis et al., 2011). Thus, the analysis in this study was based on our retrospective analysis of the studies, in which we provided a narrative and our reflections on the study program from 2003 until the end of 2020 (Duncan, 2013). The data used in the analysis were partly based on our earlier studies and publications, but the current study mainly relied on our own recollection of the CEs and reflections on the circumstances during the events. Thus, the main data sources of the study were the researchers themselves (Ellis et al., 2011). The first author has been the coordinator, student supervisor, and course instructor on the program since 2007. The second author was the leading academic of the study program between 2004 and 2015, and he has been a supervisor for most of the graduates. The CEs that we identified are described in detail in the following paragraphs.

#### CE 1: interest in a novel educational technology initiative in Finland, 2003–2004

The design of the program started at the beginning of millennium when educational technology was identified as one of the potential future research fields in Finland. An important instrument was the Finnish virtual university project, which aimed to support the use of learning technologies in Finnish universities as well to enhance collaboration between higher education institutions to establish online courses or study programs (Kähkönen & Siikelä, 2005). At the time, educational technology research and development work had already been cultivated for several years both in computer science and education within the host university. However, the host university was reluctant to provide financial support to the novel multidisciplinary initiative, which did not fit into its administrative structure. Finally, there were indications that outside funding would be available for novel initiatives in educational technology. Thus, the team of scholars involved in the planning phase launched a multidisciplinary doctoral program that would offer Finnish doctoral training especially for students from emerging and transitional countries. The first CE paved the way for the initial planning phase, which eventually resulted in an application for external funding to launch the program.

#### CE2: external seed funding, 2004–2007

The program received outside funding in 2004–2007, which enabled it to be launched and piloted. The funding also allowed project personnel to be hired to plan and design the program. Furthermore, external academics and experts were invited to design and teach online courses and supervise the doctoral students. The first cohort of students was accepted to study in the program in 2005. The main activities completed with the seed funding included the design of administrative procedures, creation of an academic network, and implementation of online study modules together with content matter experts (Hartikainen et al., 2006). Moreover, several face-to-face activities and events were organized, such as summer schools, which provided the doctoral students with opportunities to meet each other and receive face-to-face supervision. During the first years, the growth of the program was moderate (see Fig. 3). On average, during the seed funding period, three to four new doctoral students per year were accepted to start their studies. Despite the aim of launching an international study program, most of the doctoral students came from Finland. The seed funding mechanism required the activities of the program to be geared toward local students, which meant that the international aspects were not emphasized.

#### CE3: departure of a key person and the end of external funding, 2007–2008

The third CE consisted of two separate subevents that occurred very close to each other. First, in 2007 a key person left the study program, which created uncertainty for the future of the studies. Second, in the following year, the external funding ended, which had a major impact on the program’s implementation. The program had been planned to be self-sustainable after the seed funding so that the host university would allocate resources according to the number of completed doctoral degrees. However, the program did not receive any direct funding, but outcomes of the program were included in the funding of the host department. The two events caused a genuine threat to the continuation of the program, since both the financial and human resources available for running the studies decreased considerably. For example, it was no longer possible to hire full-time personnel nor to invite external experts to work on the studies. Moreover, due to the decreased financial resources, the staff members of the research group responsible for the program were required to include the activities of the program on top of their other work duties.

Hence, the structure of the program had to be renewed to ensure the continuation of the studies. The main actions for enabling the program’s continuation were to decrease the number of online courses, increase individually completed course work, and integrate the application process into the general admission process of the host university. The redesign of the studies was also supported by evaluating the experiences of the doctoral students (Paliktzoglou et al., 2010). The evaluation revealed that the students felt frustrated regarding their expectations of as well as assumptions about communication from their supervisors. A clear difference existed in the overall learning experience between those studying on campus and those participating from a distance, indicating the need to support online supervision. In addition, the students clearly indicated that they required more community support. Despite the challenging situation, the program continued to attract new students, as seen in Fig. 3.

#### CE4: expansion of interest and start of international collaboration, 2012–2014

In 2012–2013, interest in the program increased considerably compared with previous years, which caused the emergence of the fourth CE, *expansion of interest*. Despite limited resources, the intake of students increased considerably during 2012–2014, as seen in Fig. 2. A crucial milestone was reached in 2013 when the first Africa-based student graduated from the program. In 2014, the program was expanded by launching a doctoral studies hub in Tanzania in close collaboration with a local higher education institution (Apiola et al., 2015). The collaboration also involved additional financial resources, which enabled the host university to hire new staff members to supervise doctoral candidates of the hub, and also to implement teaching and courses aimed at supporting the doctoral students locally (Apiola et al., 2020). In addition, several faculty members were able to travel to Tanzania to provide intense supervision and organize face-to-face training. After 6 years of operation, four graduations have occurred through the doctoral training hub, and four doctoral students continue their studies with various context-oriented research topics.

#### CE5: personnel changes and a decrease in the work force, 2015–2017

Between 2015 and 2017, there were significant personnel changes in the program. First, a key faculty member who had been involved from the beginning and was supervising several doctoral candidates moved to another university, which caused uncertainty regarding the future of the program. Due to the unclear situation, the admission of new applicants was temporarily suspended. Furthermore, six doctoral candidates decided either to terminate their studies or to move to another university together with the former faculty member. A new staff member joined the program at the beginning of 2017, so for almost 2 years a significant decrease occurred in the work force. On the other hand, the arrival of a new staff member created opportunities to renew the program, which was clearly a positive aspect for the sustainability of the program.

#### CE6: personnel issues, collaboration challenges, and the COVID-19 pandemic, 2019–2021

The final CE included three subevents that occurred during 2019–2021. First, a key faculty member was unable to work full-time for a long period in 2019. Again, the admission of new doctoral candidates was temporarily suspended, and fewer online courses were available in the program. The second event was related to collaboration challenges with the Tanzanian higher education institution, partly due to financial issues. For example, the faculty members were no longer able to travel to Tanzania for intensive supervision and teaching. The third event was the COVID-19 pandemic, which also affected the running of the program. All campus-based activities were moved to online environments and campus visits were canceled. However, for the first time, the public examination of a doctoral dissertation was successfully held online using the Zoom video conferencing system in April 2020. Since then several examinations have been held online using either the Zoom or LifeSizeCloud online conferencing systems. In the end of 2021, a collaboration was launched with Ugandan higher education institution with the aim of launching a similar doctoral studies hub than in Tanzania.

### Analysis of the critical events

**Fig. 5:**
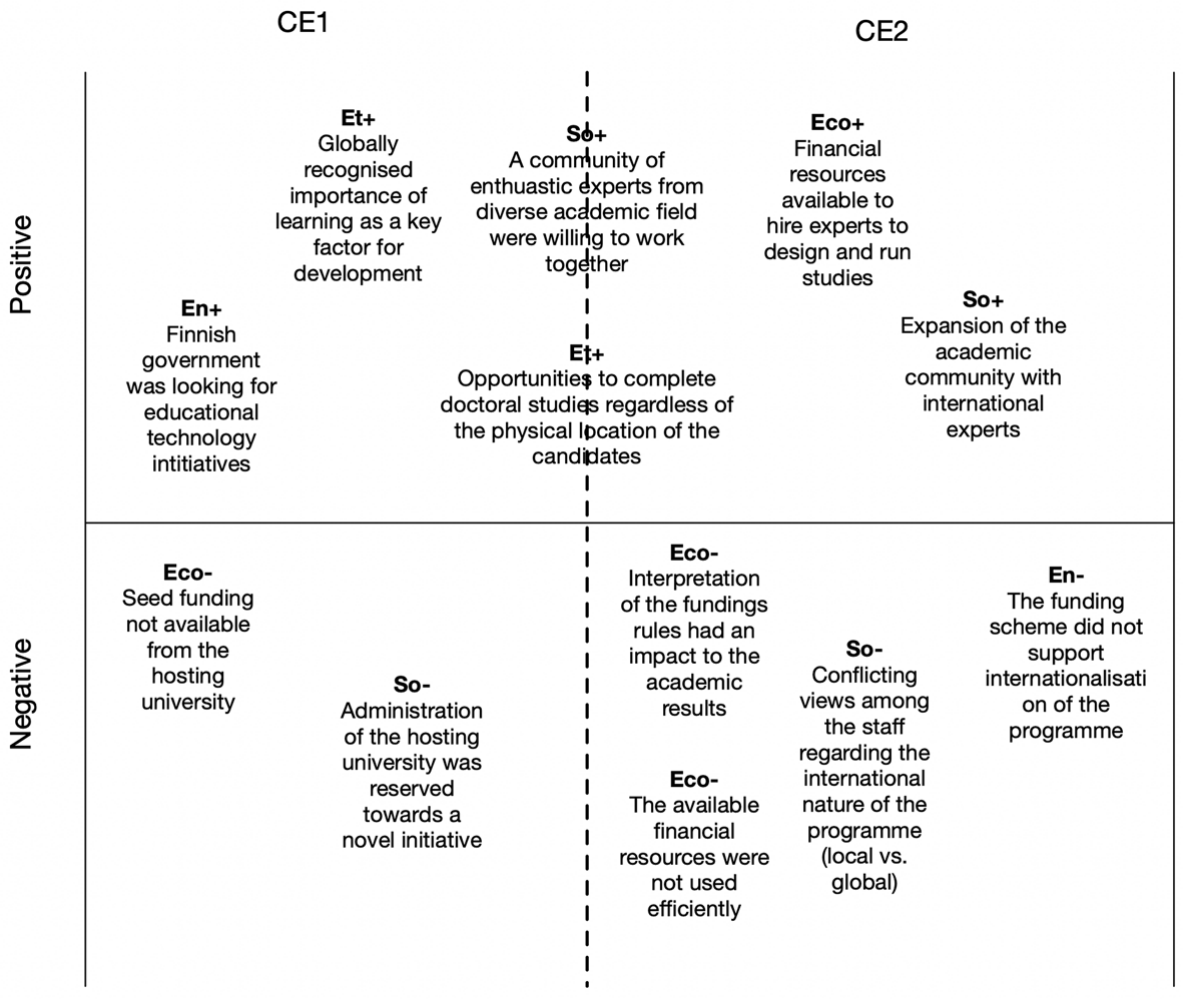
Pivotal factors for critical events 1 and 2

**Fig. 6:**
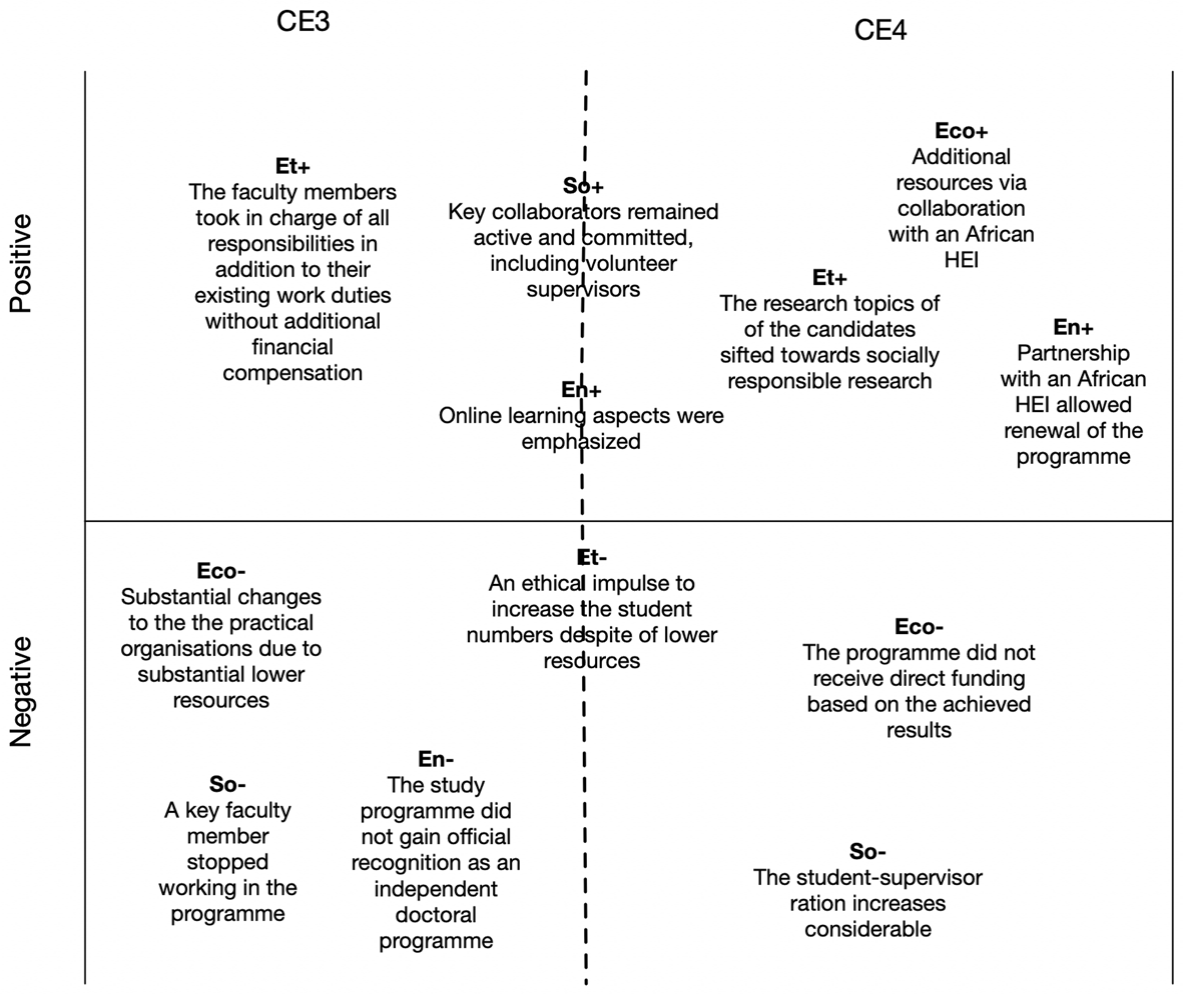
Pivotal factors for critical events 3 and 4

**Fig. 7:**
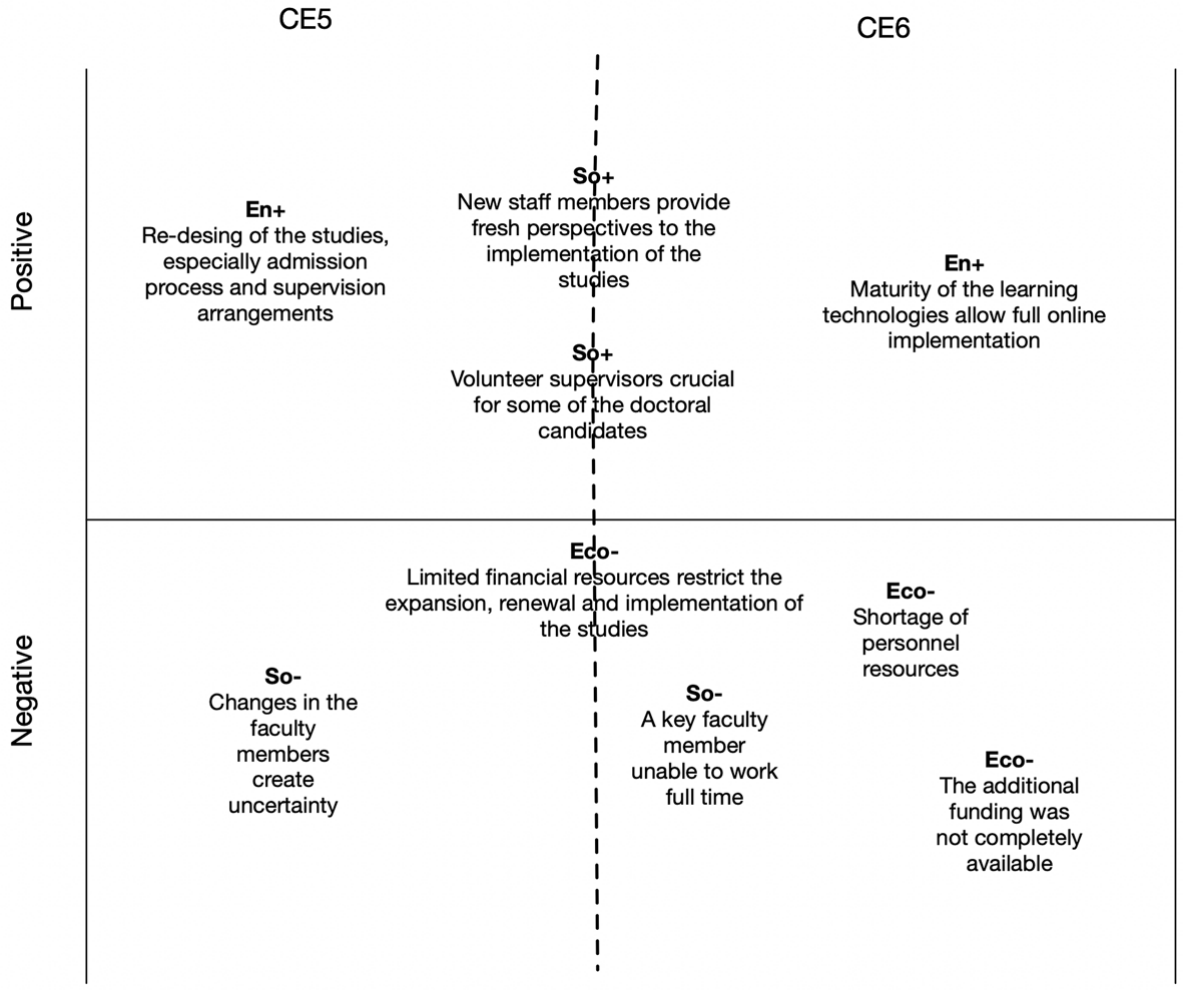
Pivotal factors for critical events 5 and 6

Figures 5, 6 and 7 present our analyses of the six identified CEs using the four-pillar sustainability model. In the analyses, we identified a set of pivotal factors related to each CE from the perspective of the sustainability pillars. The factors related to the pillars are labeled as follows: Eco+ = a positive economic factor; Eco- = a negative economic factor; Env+ = a positive environmental factor; Env- = a negative environmental factor; So+ = a positive social factor; So- = a negative social factor; Et+ = a positive ethical factor; and Et- = a negative ethical factor.

The two pivotal factors connected to CE1 as well as CE2 were especially important. At the beginning of the studies, a crucial aspect was the enthusiastic pioneers who were willing to start a completely new approach to implementing doctoral training at the host university. Furthermore, already from the beginning, a critical ethical factor was that doctoral studies could be completed through distance learning. The CEs were mainly positive in nature, but the negative factors for CE1 and CE2 had impacts on the design, implementation, and running of the studies. For example, the conflicting views about the international nature of the program strongly contradicted the original leading idea of providing studies mainly to students from emerging and transitional countries.

CE3 was highly challenging for the continuation of the studies. In a very short time frame (2007–2008), both the financial resources and human capital for running the studies decreased considerably. Although the study program survived these difficult times, major changes to the practical arrangements were required, including the reduction of online courses and supervision arrangements. For several years, the program remained active and relatively stable in terms of student numbers, but the expansion of interest in the program led to challenges since the number of staff members and local supervisors did not increase. However, collaboration with the Tanzanian higher education institution provided additional financial resources and created opportunities to renew the studies. Another crucial aspect was outside supervisors who were committed to supervising the doctoral students without any financial compensation.

CE5 and CE6 were less threatening to the sustainability of the study program compared with earlier CEs, but several aspects challenged the running of the studies. For example, due to a shortage of staff members, the admission of new doctoral candidates was suspended for long periods of time. The increase in the total number of students was moderate due to these suspension periods, but acceptance criteria were also tightened. On the other hand, for example, the maturity of learning technologies has allowed the full online implementation of the studies, which was crucial during the COVID-19 pandemic. Collaboration with two African universities has created new opportunities to supervise students and organize local support for those students who have been studying via the doctoral studies hubs.

## Lessons learned as guidelines for practitioners

Based on our analyses, we developed the following guidelines for the design and implementation of online doctoral study programs. From an economic perspective, the most interesting observation was that a good economic situation can lead to the insufficient use of resources. On the other hand, declining funding can force designers to focus on the core aspects of the studies, which can lead to improvement. The social and ethical pillars were especially crucial when the funding decreased. Despite the economic and environmental challenges, the faculty members at the host university were resilient and decided to continue running the studies.

The lessons learned from the social and ethical perspectives were the ability to redistribute responsibilities, mutual trust among faculty members, and personal ownership toward the studies, as identified previously by Russel (2009). However, one of the success factors of the doctoral study program has been the organic, not organized, and flexible involvement of a larger learning community in supervising individual doctoral students and sharing administrative duties. The learning community has been committed to taking turns and assuming complementary roles. Shared responsibility and resilience are also examples of the significance and integration of the ethical dimension for the program’s overall sustainability.

From the environmental and ethical perspectives, the role of external supervisors has been critical throughout the studies. Without their voluntary commitment, it would have been impossible to keep the student numbers as high as they have been, especially during 2016–2020. There have been two main incentives for the external supervisors: (1) an ethical motivation or internal obligation to find a doctoral training opportunity for candidates who require flexible study possibilities without study fees; and (2) the co-authored papers and eventually supervised doctoral dissertations are crucial scientific outputs that could potentially support the careers of voluntary supervisors. If a study program needs to find a voluntary work force, such as when economic resources are scarce, it is crucial to consider incentives that can motivate those volunteers. For some individuals, ethical aspects and internal motivation without financial compensation might be sufficient, but in the long run other incentives will be required.

## Discussion and conclusion

In this study, we analyzed the CEs of a Finnish online doctoral study program using the four-pillar sustainability model. We demonstrated that the model can be used to identify positive (constructive) and negative (destructive) pivotal factors related to the sustainability of online study programs from the four aspects of the economy, sociology, environment, and ethics. We claim that the proposed model can be used as a tool for the design, quality assurance, and improvement mechanisms of online study programs, since it can be used to foresee and detect potential strong and weak points. The model can also be used for identifying sustainability aspects of novel projects and initiatives; for example, it can be applied for the analysis of scalability or used for rationalizing activities.

This work was based on analyzing the CEs retrospectively. The question for future research is as follows: How can weak signs of possible fragile structures and strong points in online study programs be identified? We can also view CEs as risks that have materialized for better or worse. Thus, the four-pillar model equipped with CE and pivotal factor analysis can be used for risk management. For example, the proposed model can be used to create stress tests for an online program in the form of simulations or scenarios. These stress tests could also be supported by a formal risk analysis conducted to identify potential future CEs (Vesper et al., 2016). Other questions to consider are as follows: What is the worst-case scenario and how could it be avoided? In the best-case scenario, how can a university be convinced to take risks and operate in new ways? It is also crucial to think about the potential risks in a constructive manner when one does not know exactly what will happen. Thus, sustainable design thinking that considers all four pillars could be integrated into the design process of online study programs, such as whether the design should consider possible future use cases (Bjögvinsson et al., 2012). We also recommend that future studies propose and develop novel approaches and solutions that will help the designers and coordinators of online study programs to map out upcoming CEs and plan how to proactively react to them. Lastly, the rich data from the online program’s operation provide information that can be mined to build a model for identifying upcoming CEs, which would be an interesting challenge for learning analytics research.
